# The architecture of corneal stromal striae on optical coherence tomography and histology in an animal model and in humans

**DOI:** 10.1038/s41598-020-76963-w

**Published:** 2020-11-16

**Authors:** Pietro Emanuele Napoli, Matteo Nioi, Ernesto d’Aloja, Francesco Loy, Maurizio Fossarello

**Affiliations:** 1 San Giovanni di Dio Hospital, Clinica Oculistica, Azienda Ospedaliera Universitaria di Cagliari, via Ospedale 46, 09124 Cagliari, Italy; 2 Department of Surgical Sciences, Eye Clinic, University of Cagliari, Cagliari, Italy; 3 Department of Medical Sciences and Public Health, University of Cagliari, Cagliari, Italy; 4 Department of Biomedical Sciences, Section of Cytomorphology, University of Cagliari, Cagliari, Italy

**Keywords:** Biological techniques, Imaging, Optical imaging

## Abstract

The purpose of this study was to use a portable optical coherence tomography (OCT) for characterization of corneal stromal striae (CSS) in an ovine animal model and human corneas with histological correlation, in order to evaluate their architectural pattern by image analysis. Forty-six eyes from female adult sheep (older than 2 years), and 12 human corneas, were included in our study. The eyes were examined in situ by a portable OCT, without enucleation. All OCT scans were performed immediately after death, and then the eyes were delivered to a qualified histology laboratory. In the ovine animal model, CSS were detected with OCT in 89.1% (41/46) of individual scans and in 93.4% (43/46) of histological slices. In human corneas, CSS were found in 58.3% (7/12) of cases. In both corneal types, CSS appeared as “V”- or “X”-shaped structures, with very similar angle values of 70.8° ± 4° on OCT images and 71° ± 4° on histological slices (p ≤ 0.01). Data analysis demonstrated an excellent degree of reproducibility and inter-rater reliability of measurements (p < 0.001). The present study demonstrated that by using a portable OCT device, CSS can be visualized in ovine and human corneas. This finding suggests their generalized presence in various mammals. The frequent observation, close to 60%, of such collagen texture in the corneal stroma, similar to a ‘truss bridge’ design, permits to presume that it plays an important structural role, aimed to distribute tensile and compressive forces in various directions, conferring *resilience* properties to the cornea.

## Introduction

Corneal stromal striae (CSS) are fine colorless lines in the central cornea that depart from Descemet’s membrane and extend in a vertical or near-vertical direction to end in the mid or anterior stroma, or even Bowman’s layer. They were first described as glassy corneal striae (GCS) by Sturrock in 1973 in normal human corneas by means of slit-lamp biomicroscopy^1^. Histologically, CSS are represented by undulations in continuous lamellae composed of collagen VI, keratocan and lumican^2^.

CSS differ from other corneal striae such as Vogt’s striae observed in keratoconus, vertical striae associated with contact lens-induced corneal edema, diabetes-associated striae, and idiopathic Descemet’s membrane wrinkles, striae associated with LASIK flap or SMILE Lasik technique, all of which also are vertical in orientation, but lacking a criss-cross design. However, GCS do not disappear with increased intraocular pressure induced by digital palpation of the globe, as do the other described striae^3^.

A hypothetical role of CSS is to maintain a normal corneal contour and providing stability to the cornea, in order to reduce the mechanical corneal stress due to external and/or internal traumatic forces^2,3^.

In the last years, optical coherence tomography (OCT) technology has been widely applied to study human cornea in vivo and ex vivo. In a recent study of Grieve and coll., CSS have been described in humans and in two other mammals, macaque and mouse, by means of histology, scanning electron microscopy, OCT, and full-field optical coherence tomography (FFOCT)^2^.

We have recently demonstrated the reliability of portable OCT imaging in studying the human and ovine corneas, both in clinical and in experimental setting^4–9^.

The purpose of this study was to use portable OCT for characterization of CSS in an ovine animal model (i.e. *Ovis aries*) and human corneas with histological correlation, in order to evaluate their architectural pattern by image analysis.

## Methods

Forty-six eyes from female adult sheep (older than 2 years), sacrificed at a local slaughterhouse, were utilized in our study. After animal decapitation, the eyes were examined by OCT in situ, without enucleation. The instrumental analysis was performed using a portable spectral-domain OCT (SD-OCT) system (iVue SD-OCT, Optovue Inc, Fremont, CA). This OCT system works at a frame rate of 256–1024 A-scan/frame, with an image acquisition rate of 26,000 axial scans per second, and has a 5-μm axial resolution. This SD imaging machine uses a center wavelength of 840 ± 10 nm to provide high-resolution scans.

As previously reported, all examinations were conducted in the same conditions of temperature (within a range of 12–22 °C) and humidity (within a range of 50–60%)^10,11^.

All OCT scans were performed immediately after death (in a time window of less than 15 min). Then, the eyes were assigned to a qualified histology laboratory.

**Figure 1:**
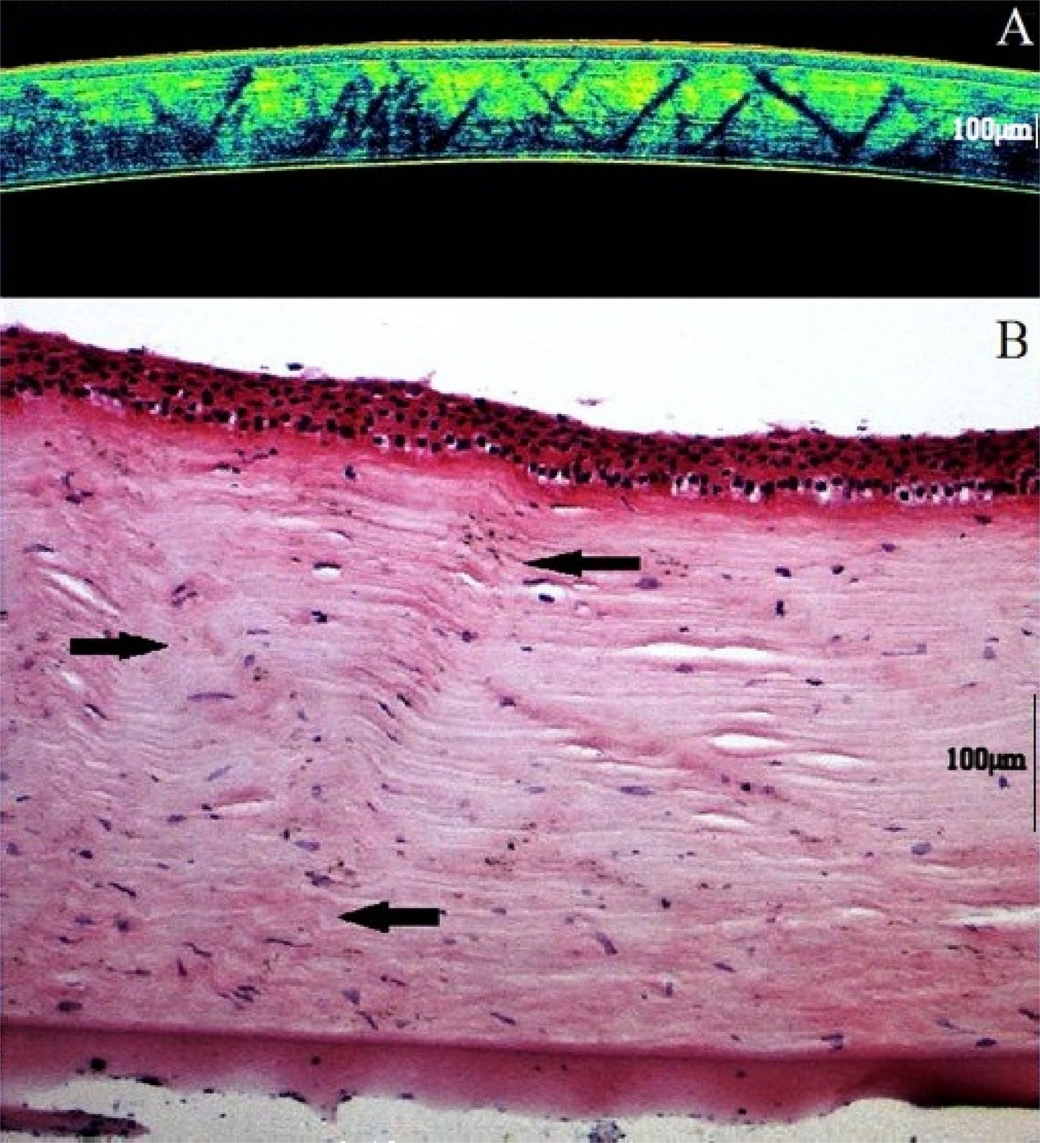
Optical coherence tomography image (**A**) of corneal stromal striae obtained by in situ scanning of our animal model (resembling a ‘truss bridge’ design). (**B**) Histological image (hematoxylin–eosin ×40) of the same cornea (the arrows indicate the “X-shaped” stromal structures) (Scale bar = 100 µm).

In order to obtain a minimal tearing, folding and/or the introduction of other artifacts, the corneas were carefully cut and spread in the water bath and properly floated onto a microscope glass slide. Thereafter, the corneas were stained with hematoxylin and eosin and scanned at 40× (standard 24-bit RGB camera) (Fig. 1). All procedures on animals were in accordance with The Faculty of Chemical Science Animal Research Act and the Association for Research in Vision and Ophthalmology (ARVO), Statement for the Use of Animals in Ophthalmic and Vision Research.

**Figure 2:**
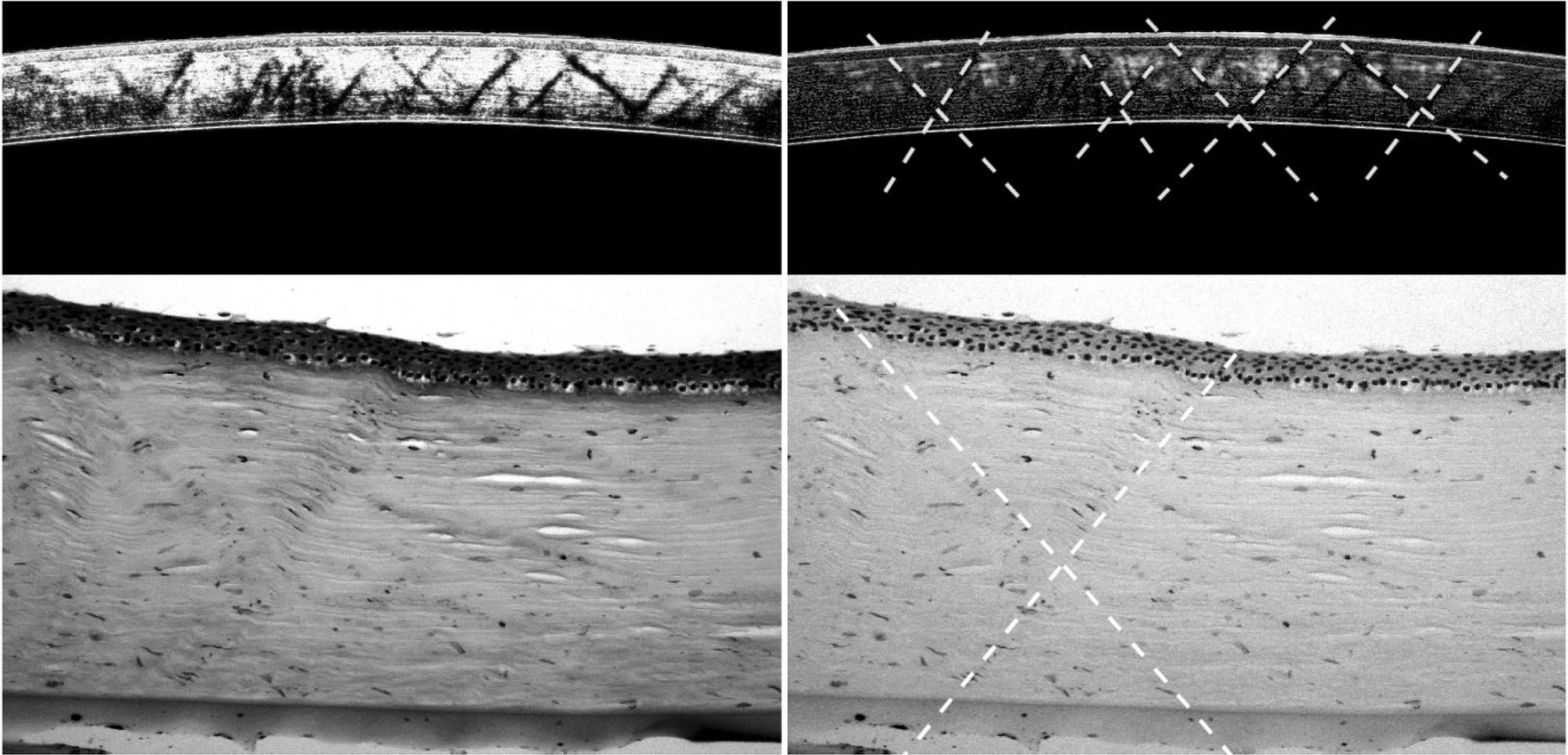
Quantitative analysis of angles within the geometric stromal striae. Grayscale images obtained by splitting the original picture into the green (left) and red (right) channels, which provide the best contrasts to explain how angles have been defined. Optical coherence tomography scans (top) and histological slides (bottom) are reported without (left) and with (right) the placement of markers for measurement of *θ*_A_ for *θ*_B_ angles, respectively (see text). The elements of the criss-crossing *superstructure* are defined by two continuous, oblique lines ("V"- or "X"-shaped) in the stromal tissue, which are indicated by the dashed lines. For the purposes of our analysis, since the vertically opposite angles (i.e. the upper and the lower) have identical (or congruent) values, only one of these was indiscriminately included in the data collection (according to the *vertical angle theorem)*. Conversely, the lateral ones (or adjacent angles) were not considered in our computation. Of note, the various angles within the individual corneas have demonstrated to be equal, or approximately equal, in measure.

Our analysis was focused on the central cornea (0–3 mm on OCT, and 0–3 mm on histology), considering the vertical plane described from the 12th to the 6th clock hour, from a cross-sectional point of view. Corneal image analysis was performed by *ImageJ* software (ImageJ version 1.52p, National Institutes of Health, USA; available at: https://imagej.nih.gov/ij/) to quantify the angles within *geometric structures* (or patterns) delineated by stromal striae on OCT scans (*θ*_A_) and upon histological slides (*θ*_B_) (Fig. 2).

Specifically, the angle (*θ*) has been defined as the angle formed by two linear stromal striae describing a flat figure, in the shape of the Latin letter V or X: an oblique line between DM to BL, and a contiguous/intersecting line with an approximately specular path.

In our analysis, since the two *vertically opposite angles* (i.e. the upper and the lower) have identical (or congruent) values according to the *vertical angle theorem*, only one of these was indiscriminately included in data collection. Conversely, the lateral ones (or adjacent angles) were not considered in our computation.

All images were exported and processed for the quantitative analysis by two operators (PEN, MN).

**Figure 3:**
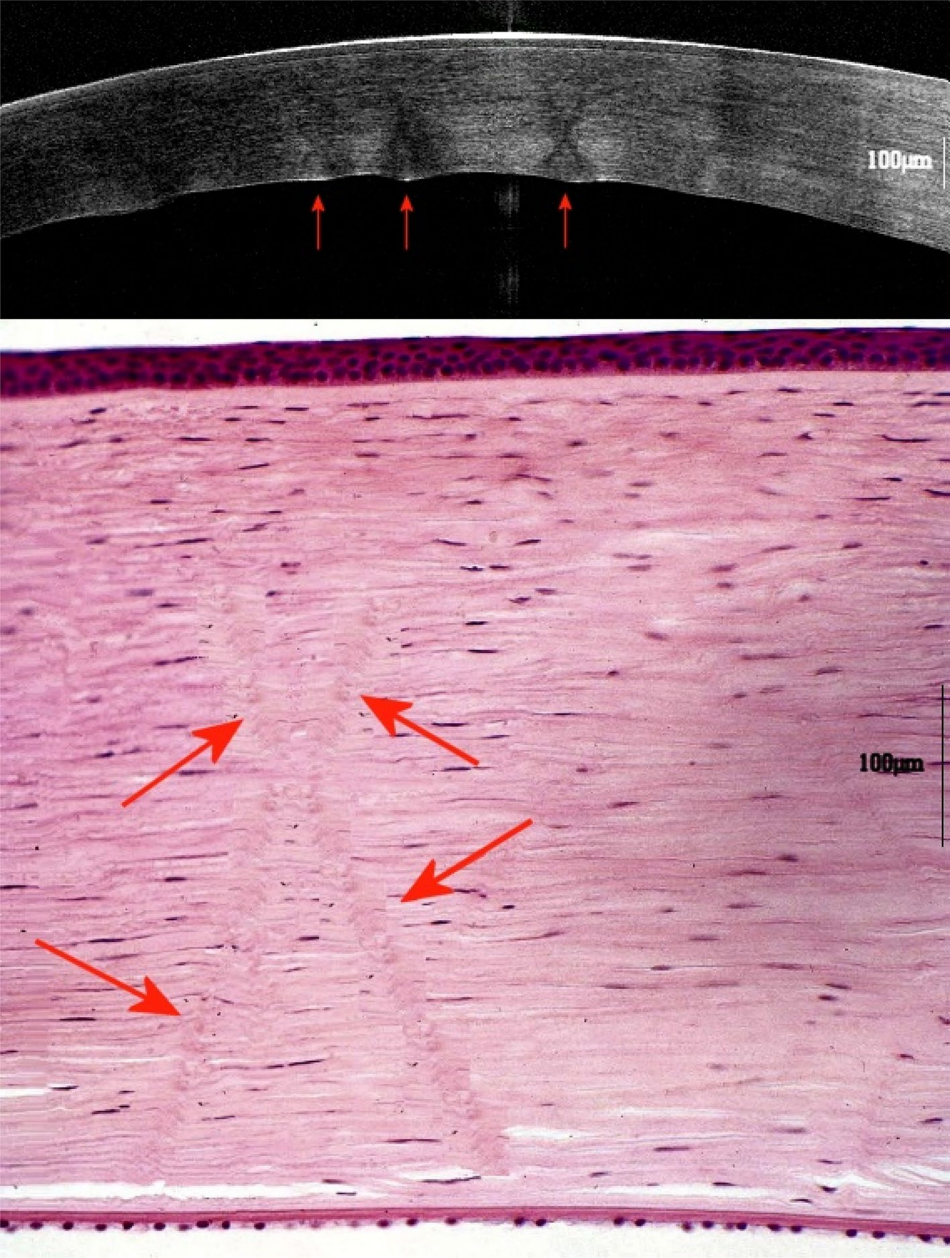
Corneal stromal striae in human cornea on optical coherence tomography imaging (top) and histology (bottom) (grayscale and color images). The arrows indicate the most easily detectable “X-shaped” linear structures (Scale bar = 100 µm).

The overall results were also compared with those relating to 12 human corneas, which were obtained from coroner’s autopsies (as an integral part of normal forensic practice for decapitation, and gunshot wound to the orbit) in the early postmortem interval (Fig. 3). The present work was conducted ethically according to the principles of the Declaration of Helsinki. The protocol was previously evaluated by local Independent Ethical Committee (IEC) of the University of Cagliari that, according to local rule, considered ethical the use of the human specimens due to the non-invasive nature of OCT evaluation and the due histological examination of cornea authorized by the local general Prosecutor in charge for the single case (*Regolamento di Polizia mortuaria,* DPR 285/90). Informed consents of next of kin were obtained by appropriate district attorney.

These eyes were considered as healthy, since associated with unremarkable medical history (collected from review of past medical records) and a normal ophthalmologic examination.

## Statistical analysis

Statistical analysis was carried out using Statistical Package for Social Science SPSS version 21.0. Continuous numeric data were summarized as mean ± standard deviation (SD), and categorical data were expressed as percentages (%). Data were analyzed by Lilliefors test and Shapiro–Wilk test for normality.

The Spearman’s ρ test and Kendall’s τ test were performed to determine the relationship between the angles measured on OCT scans (*θ*_A_) and histological slides (*θ*_B_), and within the individual corneas (*θ*_1_, *θ*_2_, *θ*_3_). For the latter analysis, three geometric structures were examined in each image (*θ*_1_, *θ*_2_, *θ*_3_).

With an accepted alpha risk of 0.05 and a β risk of 0.20 (i.e., 80% statistical power) in a two-sided test, approximately 11 individual measurements (on each imaging modality) are needed before a correlation coefficient of 0.76 is said to be statistically significant.

OCT measurements obtained by one operator (MN) were used only for reproducibility analysis. Overall, the correlation between individual measurements was estimated by the intraclass correlation coefficient (ICC). p-values less than 0.05 were considered significant.

## Results

The results of OCT imaging performed by the two operators are summarized in Tables 1, 2, 3. Informative images of corneal stroma were easily obtained in all cases. Descriptive statistics such as mean, standard deviation, and percentage were calculated for each imaging modality.

**Table 1:** Descriptive data of the two imaging modalities (optical coherence tomography and histology).

	Corneal stromal striae			
	Ovine animal model		Human (healthy)^a^	
	OCT	Histology	OCT	Histology
N. of positive cases/total (percentage)	41/46 (89.1%)	43/46 (93.4%)	7/12 (58.3%)	7/12 (58.3%)
N. of geometric structures analyzed	252	252	42	42
Mean of geometric structures detected for each scan/slide (density)	10.1	9.6	5.2	5.1
**Full-thickness geometric structures (percentage)**^b^				
Number (percentage)	176/252 (69.8%)	178/252 (70.6%)	24/42 (57.1%)	26/42 (61.9%)
Overall N. (percentage)	177/252 (70.2%)		25/42 (59.5%)	
**Only posterior geometric structures**^c^				
Number (percentage)	76/252 (30.1)	74/252 (29.3)	15/42 (35.7%)	17/42 (40.4%)
Overall N. (percentage)	75/252 (29.8%)		16/42 (38.0%)	
Angle values (°) [mean ± SD] *θ*_A_ and*θ*_B_	70.8 ± 4	71 ± 4.2	62.5 ± 10	69.4 ± 2.4

^a^Corpses obtained from coroner’s autopsies. These corneas were considered as *healthy*, since associated with a normal ophthalmologic examination and unremarkable medical history (collected from review of past medical records).

^b^Corneal stromal structures found to run through the entire corneal thickness (at least one line of the geometric figure).

^c^Corneal stromal structures non-touching the anterior stroma (e.g. the Bowman membrane). *θ*_A_ = Angle measured by OCT scans. *θ*_B_ = Angle measured by histological slides. *OCT *Optical coherence tomography.

**Table 2:** Intraclass Correlation Coefficients (ICCs).

	Intraclass correlation coefficient (ICC)		Significance
	Single measures	Average measures	(*p*)
**Variables**			
Angle values within the individual corneas (*θ*_1_, *θ*_2_, *θ*_3_)^a^	0.999	1.000	< 0.001
OCT (*θ*_A_)—histology (*θ*_B_)	0.858	0.924	< 0.001
**Ovine animal model—human**			
OCT (*θ*_A_)	0.792	0.862	< 0.001
Histology (*θ*_B_)	0.803	0.879	< 0.001
**Intra-test reliability (operator 1)**			
OCT (*θ*_A_)	0.998	0.999	< 0.001
Histology (*θ*_B_)	0.988	0.991	< 0.001
**Intra-test reliability (operator 2)**			
OCT (*θ*_A_)	0.994	0.995	< 0.001
Histology (*θ*_B_)	0.983	0.987	< 0.001
**Inter-test reliability (operator 1–2)**			
OCT (*θ*_A_)	0.842	0.921	< 0.001
Histology (*θ*_B_)	0.824	0.933	< 0.001

*θ* = Angle values within the architectural patterns. The angles (*θ*) have been defined as the angle formed by two linear stromal striae describing a flat figure, in the shape of the Latin letter V or X (see text). Since the two *vertically opposite angles* (i.e. the upper and the lower) have identical (or congruent) values according to the *vertical angle theorem*, only one of these was indiscriminately included in data collection.

*OCT *Optical coherence tomography.

^a^For this analysis, three geometric structures were evaluated in each image (*θ*_1_, *θ*_2_, *θ*_3_…).

**Table 3:** Correlation analysis between metric results obtained with optical coherence tomography and histology.

					Correlation coefficient		Significance
					Spearman’s rho (ρ)	Kendall’s tau (τ)	(*p*)
	**Variables**						
Ovine animal model	Angle values within the individual corneas (*θ*_1_, *θ*_2_, *θ*_3_)^a^	OCT		*θ*_1_, *θ*_2_	0.995	0.974	< 0.001
				*θ*_1_, *θ*_3_	0.994	0.975	< 0.001
				*θ*_2_, *θ*_3_	0.993	0.967	< 0.001
		Histology		*θ*_1_, *θ*_2_	0.989	0.971	< 0.001
				*θ*_1_, *θ*_3_	0.988	0.969	< 0.001
				*θ*_2_, *θ*_3_	0.990	0.971	< 0.001
	OCT (*θ*_A_)—histology (*θ*_B_)				0.872	0.775	< 0.001
Human	Angle values within the individual corneas (*θ*_1_, *θ*_2_, *θ*_3_)*	OCT		*θ*_1_, *θ*_2_	0.993	0.973	< 0.001
				*θ*_1_, *θ*_3_	0.993	0.973	< 0.001
				*θ*_2_, *θ*_3_	0.992	0.965	< 0.001
		Histology		*θ*_1_, *θ*_2_	0.985	0.966	< 0.001
				*θ*_1_, *θ*_3_	0.986	0.967	< 0.001
				*θ*_2_, *θ*_3_	0.984	0.965	< 0.001
	OCT (*θ*_A_)—histology (*θ*_B_)				0.837	0.768	< 0.01
Ovine animal model—human			OCT (*θ*_A_)		0.841	0.762	< 0.01
			Histology (*θ*_B_)		0.837	0.765	< 0.01

*θ* = Angle values within the architectural patterns (see text).

*OCT *Optical coherence tomography.

^a^For this analysis, three angles were evaluated for each image (*θ*_1_, *θ*_2_, *θ*_3_…).

In the animal model, the geometric structures were detected on OCT and upon microscopic examination in 89.1% (41/46) and in 93.4% (43/46) of cases, respectively. Overall, they were found to run through the entire corneal thickness (at least one line of the geometric figure) in 70.2% of the cases. Specifically, *θ*_A_ and *θ*_B_ values were of 70.8° ± 4° and 71° ± 4°, respectively.

In human corneas CSS were observed on OCT and microscopically in 58.3% (7/12) of cases. However in these corneas CSS show lower density and steeper angles (Table 1).

Furthermore, only 59.5% of these were found to run through the whole thickness, being primarily represented in the posterior stroma.

Of note, the various angles within the individual corneas (*θ*_1_, *θ*_2_, *θ*_3_) demonstrated to be equal, or approximately equal, in measure (Spearman’s ρ ≥ 0.984, p < 0.001; Kendall’s τ = 0.965, p < 0.001; ICC ≥ 0.99, p < 0.001). Moreover, a highly significant correlation was found between angle values detected by OCT and histology (Spearman’s ρ ≥ 0.837, p < 0.001; Kendall’s τ ≥ 0.768, p < 0.001; ICC ≥ 0.86, p ≤ 0.01).

Statistically significant ICC values (p < 0.001) were found for angle size measurements in the two separate datasets, demonstrating the excellent inter-rater reliability and reproducibility of results.

## Discussion

The data of our study demonstrated the existence of CSS in a new animal model (i.e. *Ovis aries*), thus suggesting their ubiquity in different mammalian species. The presence of these previously unappreciated corneal structures in different animal species (i.e. humans, macaques, rabbits and sheep) implies their crucial role in corneal physiology and biomechanics. As previously reported, this structural organization of stromal tissue may be important to reduce and counteract corneal stresses, such as it may happen for instance during external shocks or the spikes of intraocular pressure.

In the present work, OCT imaging revealed the stromal striae as regular, criss-cross lines of hypo-reflectivity departing from DL to BM. OCT scanning session proved to be simple, fast and easily feasible at the study site (since it is relatively lightweight and transportable). Therefore, the portable OCT system has confirmed its high inter-rater reliability for experimental studies on samples examined by different operators in the early postmortem period^12^. Specifically, our novel approach suggested that it is possible to analyze in vivo the tissue texture and biomechanical features of the cornea in a non-invasive way by OCT imaging, and to use the ovine animal model for preliminary experiments (for example to verify that some treatment has a potential in modifying the functions and structures of the cornea in situ).

Compared to human corneas, the “V”- and “X”-shaped stromal structures detected in ovine corneas generally showed flatter angles (also obtuse angles) and larger interwoven arrangements, suggesting that the latter tissue may be used for easier pattern recognition in the structural analysis due to the larger size and higher density of its geometric elements.

In accordance with previous studies, in human samples we found a large percentage of geometric CSS only in the posterior part of stroma, not reaching the BM^1–3^. This implies that the mechanical properties of this heterogeneous viscoelastic tissue, in particular its resilience, may vary considerably as a function of depth, i.e. of its structural design. As a matter of fact, several studies on the biomechanics of human and rabbit corneas, indicate that anterior and posterior stroma are characterized by significant differences in elastic modulus (known as Young’s modulus)^13–15^. Moreover, several clinical experiments have shown that corneal crosslinking, a medical procedure employed to increase the stromal stiffness in case of corneal ectatic pathologies, may stop the progression of the structural distortion, despite acting mainly on the anterior stroma^16–19^. All these data confirm that anterior and posterior portion of the stroma have different biomechanical properties, which perform diverse specific functions.

Overall, in our animal model, the CSS were detected on OCT and upon microscopic examination in 89.1% (41/46) and in 93.4% (43/46) of cases, respectively, while in humans in 58.3% (7/12) of cases (Table 1). These percentages are slightly higher than those found by other Authors^2,3^. The larger number of CSS observed at microscopic examination is essentially due to the smaller window of OCT imaging, which detects only a portion of the total field.

Noteworthy, the various angles within the individual corneas appear equal, or approximately equal, to each other. This explains the high correlation between the results obtained with the two imaging methods beyond the possibility of having considered slightly different corneal sections.

These structural findings, demonstrating a regular geometric texture of CSS, similar to that of a ‘truss bridge’, suggest their crucial role in maintaining the shape of the cornea and visual acuity. In fact, as in the case of ‘truss bridges’, the triangular units (Fig. 2) allow the *superstructure* to effectively tolerate stresses from tensions, compressions, or sometimes both, in response to dynamic loads^20^. It is well known that a structural design based on similar connected elements implies the ability to distribute the forces in various directions (conferring *resilience* properties rather than biomechanical stiffness)^20^. Specifically, resilience is the ability of the tissue to absorb energy whn deformed, and to release this energy when discharged. Although previous studies revealed that tensile strength is associated with collagen VI^21^, no protein had ever been found so far to explain corneal elasticity^2,22^.

It is interesting to note that in corneal pathologic conditions, such as keratoconus, CSS appear different, showing a more parallel (not criss-cross) arrangement. Therefore, CSS should be further explored in the future to better understand which factors most contribute to corneal ectatic disorders.

Interestingly, an important peculiarity of the human and ovine cornea is the nearly absent replication rate of endothelium cells, a characteristic that is completely different in rodents^23^. Consequently, the ovine cornea should be considered more suitable than that of rodents and macaques, not only for its histology and easy availability, but also for its immunological characteristics^24^.

In conclusion, the results of the present study indicates that CSS can be reliably studied by a portable OCT in an ovine model, and that it represents a more accessible and valuable analogue of human cornea.
